# Turkish Society of Gastroenterology for United European Gastroenterology: A Mutual Relationship

**DOI:** 10.5152/tjg.2024.231903

**Published:** 2024-04-01

**Authors:** J. Matthias Löhr

**Affiliations:** 1 President, United European Gastroenterology, Center for Digestive Diseases, Karolinska University Hospital, Stockholm, Sweden; 2 Department of Clinical Science, Intervention and Technology (CLINTEC), Karolinska Institute, Stockholm, Sweden

Turkish Society of Gastroenterology (TSG) is a member of the United European Gastroenterology (UEG). As one of 49 national societies, Turkey is represented in the National Societies Forum of UEG who in turn sends delegates to the “Meeting of Members” (MoM), the parliament of UEG (Figure 1). From here delegates are sent to the Council, the government of UEG. The council is presided by the “Executive Committee” (ExCom), consisting of the president, vice-president (president-elect), the general secretary, and the treasurer. They all are elected by the MoM. The other way in is via one of the 17 European specialty societies such as EASL, EDS, EPC, ESGE, ESNM, to name a few, that also send delegates to the MoM and in turn to Council. Council then votes on the chairs of five committees and three groups: Scientific, Education, Quality-of-Care, Research, and National Societies Committees as well as the Public Affairs, Equality & Diversity and, last but not least the Young Talent Group (Figure 1). All committees are staffed by delegates sent from the Ordinary member societies. Members to the three groups are recruited by recommendation and/or suggestions from the group themselves.

The groups and committees work independent, i.e. Council or ExCom are not influencing their agenda. The Scientific Committee deals with the program for the UEG Week, today the centerpiece of the UEG activities, being the largest GI meeting in the world. The Education Committee is delivering and disseminating cutting-edge, unbiased continuing medical education (CME) and supporting professional development.^1^ The Research Committee is encouraging innovation and excellence in research by facilitation and cooperation, e.g. participating in EU projects. The Quality-of-Care committee is striving to improve and harmonise patient care and practices across Europe: evidence-based guidelines, offered in the complimentary GI Guidelines app, are central in this regard. The voice and issues of National Societies are represented in each Committee through cross-representatives from the National Societies Committee. The Public Affairs Group is actively lobbying in Brussels as the united voice of European Gastroenterology for all topics within our major theme, i.e. Digestive Health.^2^ Equality & Diversity Group promotes exactly that within UEG but also for all of European Gastroenterology.^3^ Finally, the Young Talent Group has turned out to become the rejuvenating fountain by recruits from the Young talent pool where everybody can sign up (via myUEG), present themselves and offer their desired service to our federation.

The UEG Journal represents another feather in our cap: it has evolved in one of the best GI journals with 20k followers on social media and IF of 6. It is completely free access. From the Young Talent pool, junior editors were recruited to learn the business of reviewing and running a journal under the wings of the seasoned editorial team.

Why is UEG of any interest to TSG? For the young gastroenterologists, it offers a chance to engage in interesting, even challenging work to shape the future of European Gastroenterology, in the Young Talent Group, UEG Journal and other committees. For those who represent SGF in any of the committees, our voice becomes heard. UEG offers travel grants to those attending UEG Week, short-time visits to hospitals or research groups in Europe, research stipends, postdoctoral awards, and a research prize. By becoming a myUEG Associate, all of the educational content including the recorded lectures from UEG Week as well as all other online webinars can be accessed via myUEG – a platform one can sign up for free.^4^ This includes videos from endoscopy as well as a library of pictures (“image hub”) for educational purposes. Once signed up, one will receive information on all ongoing activities within UEG – online and face-to-face. Within this platform and the “my Connect” feature, one can form an online forum for any purpose or search for a colleague with a special interest or knowledge. UEG also offers meeting rooms for so-called common interest groups that will receive time and space during an UEG Week to physically meet.

Turkey/TSG fared well so far with UEG participating in several committees and receiving an activity grant for last year’s Annual Meeting. TSG has participated in the first ever^5^ and two more evidence-based guidelines^6,7^ (and one more to come). Nevertheless, considering the strength of Turkish Gastroenterology, members of TSG could both make more use and engage more on the European level – to shape the future of European gastroenterology together.^8^ The landscape of education, exams,^9^ industrial support and policy will change with challenges from the environment^10^ to artificial intelligence.^11,12^ Become a myUEG Associate and evaluate the many opportunities yourself and join me in shaping the future of European gastroenterology.^13^

## Figures and Tables

**Figure 1.**
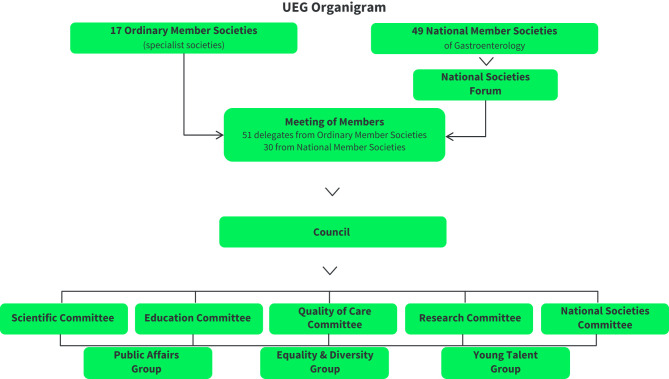
Organigram of UEG.
